# Takotsubo Cardiomyopathy After Esophageal Surgery in a Patient With Undiagnosed Coronary Artery Disease

**DOI:** 10.7759/cureus.87482

**Published:** 2025-07-07

**Authors:** Ernestine Faye S Tan, Sakar B Gharti, Mugtaba Ahmed, Yohannes Debebe Gelan, Ajibola M Adedayo

**Affiliations:** 1 Internal Medicine, Interfaith Medical Center, New York, USA; 2 Cardiology, Interfaith Medical Center, New York, USA

**Keywords:** acute coronary syndrome, cardiomyopathy, coronary artery disease, esophageal surgery, restrictive cardiomyopathy, surgery, surgical complications, takotsubo, takotsubo cardiomyopathy (ttc), thoracic surgery

## Abstract

Takotsubo cardiomyopathy (TCM), also known as "broken heart syndrome" or stress cardiomyopathy, is a transient, non-ischemic form of heart failure characterized by left ventricular apical ballooning, elevated cardiac enzymes, and regional systolic dysfunction. It commonly presents with symptoms similar to acute coronary syndrome (ACS), leading to frequent misdiagnosis. TCM is often triggered by significant emotional or physical stress, with a notable predilection in postmenopausal women. Although typically reversible, the condition's pathophysiology remains incompletely understood, with the most accepted theory linking it to excessive catecholamine release and myocardial dysfunction.

We present a case of a 73-year-old female who developed TCM following an elective right thoracotomy for esophageal cyst resection. The patient exhibited elevated cardiac enzymes and arrhythmias postoperatively. Despite an initial suspicion of ACS, further cardiac work-up, including echocardiography and cardiac catheterization, showed new-onset severe systolic dysfunction and apical ballooning characteristic of TCM. Interestingly, the patient also had an undiagnosed coronary artery disease incidentally found during cardiac catheterization. The patient's condition improved over the following months, with normalization of left ventricular function, supporting the reversible nature of TCM.

This case underscores the need for thorough evaluation when diagnosing heart failure with no clear ischemic cause, ensuring that ACS or CAD is not overlooked. While TCM can be resolved with appropriate care, careful clinical judgment is essential to avoid misdiagnosis and optimize patient outcomes.

## Introduction

Takotsubo cardiomyopathy (TCM), also known as “stress cardiomyopathy,” is a form of non-ischemic cardiomyopathy characterized by transient apical ballooning, elevated cardiac enzymes, and regional systolic dysfunction [1,2]. It is commonly misclassified as acute coronary syndrome (ACS) due to the similar clinical and diagnostic presentation, although the former is a form of reversible, non-ischemic heart failure that resolves completely in one to six months [2]. 

Cases have been reported to predominantly affect postmenopausal women and are often related to life events that cause significant physical or emotional strain, such as abuse, deaths of relatives, calamities, accidents, medical procedures or illnesses, and stimulant drugs [1,3,4]. A report by the International Takotsubo Registry showed that the rates of neurologic or psychiatric disorders were higher in patients with TCM than those with ACS (55.8% vs. 25.7%, with a p <0.001) [3].

Despite TCM being commonly described as the “broken heart syndrome” in literature, it may also be triggered by significant positive life events, and in other cases, it may have no trigger at all [3]. Epidemiological reports reveal that TCM accounts for 1-3% of ACS and 0.5% to 0.9% of ST-segment elevation myocardial infarcts [5,6], although cases remain to be underreported or misclassified as ACS due to the similarity in presentation. Coronary angiography with ventriculography remains the gold standard for definitive diagnosis.

Diagnosing TCM in the presence of coronary artery disease (CAD) is challenging due to overlapping clinical features with ACS, including chest pain, ST-segment changes, troponin elevation, and regional wall motion abnormalities (RWMA). While TCM typically presents with RWMA that extend beyond a single coronary territory and modest troponin elevation relative to the degree of dysfunction, the presence of coexisting CAD can obscure this pattern, leading to misdiagnosis. Coronary angiography may reveal significant stenosis, prompting inappropriate revascularization or masking the true cause of myocardial dysfunction. The diagnostic complexity presents a significant challenge in avoiding misdiagnosis and guiding appropriate management.

Despite the rising cases of TCM, a consensus regarding the management remains to be developed. In hopes of further understanding this condition, we present a case of TCM in a patient who was admitted for elective right thoracotomy for esophageal cyst resection, but had a complicated postoperative course characterized by new-onset severe systolic dysfunction, apical ballooning, elevated cardiac enzymes, global hypokinesis, and unstable atrial fibrillation. 

## Case presentation

This is a case of a 73-year-old Caucasian female patient from the city of New York who was admitted for elective right thoracotomy and resection of symptomatic esophageal cysts. The patient had no significant past medical history, family history, or substance use history. 

**Figure 1:**
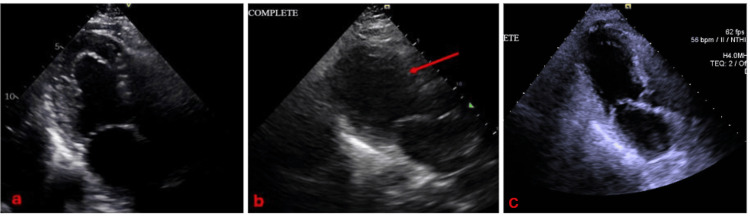
Comparison of left ventricular size before (a) and after (b) surgery, and two months post-surgery (c). A red arrow depicts new onset ballooning of the mid and apical segments of the left ventricle, raising concern for TCM. TCM: takotsubo cardiomyopathy

Prior to surgery, the patient underwent cardiac evaluation and clearance. A 2D echocardiogram showed normal systolic and diastolic functions, with an ejection fraction (EF) of 60%. The left ventricle (LV) had normal wall thickness (see Figure 1a) and normal wall motion. No other significant findings were noted. A stress test was done, which showed normal results. The patient denied any symptoms and was stratified as intermediate risk to undergo the procedure. The patient underwent the procedure with no intraoperative complications. 

**Figure 2:**
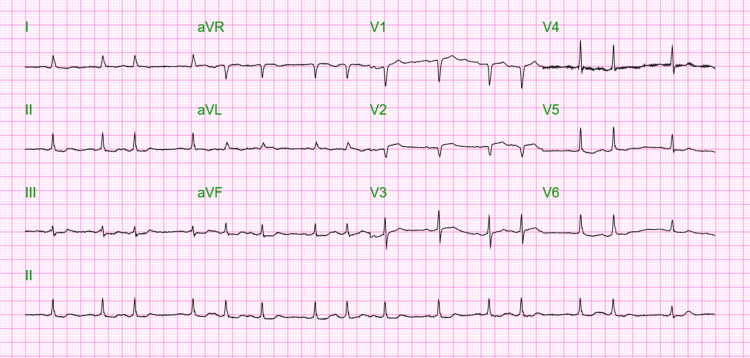
Electrocardiogram showing atrial fibrillation with controlled ventricular response after atropine administration

During the postoperative course, the patient was referred for hypotension and new-onset atrial fibrillation with slow ventricular response (47 beats per minute) noted on cardiac monitor, for which atropine was given. The patient was eventually placed on norepinephrine and epinephrine drips for persistent hypotension and bradycardia, reflecting cardiogenic shock. An electrocardiogram (EKG) was done after treatment, showing improvement of the bradycardia, although the patient remained hypotensive and in atrial fibrillation (see Figure 2). 

Troponin I was serially monitored, which increased from 1052 pg/ml to >25,000 pg/ml in less than 24 hours (normal value <11.8pg/ml). Brain natriuretic peptide (BNP) was 162 pg/ml (normal value <100 pg/ml). Electrocardiograms were serially monitored, which showed atrial fibrillation with no ST elevations or depressions. Repeat 2D echocardiogram showed a dilated LV with severely decreased systolic function and an EF of 28%. Global hypokinesis, with ballooning of mid and apical segments, was consistent with TCM (see Figure 1b). The hypotension and bradycardia improved, and vasopressors were discontinued within 24 hours. Computed tomographic pulmonary angiography was negative for pulmonary embolism. 

Diagnostic cardiac catheterization was done, which showed severe single-vessel CAD with 100% thrombotic occlusion of the large OM3 branch, severe LV dysfunction with an EF of <30%, and a severely elevated LV end-diastolic pressure of 40 mmHg. Left ventriculogram was consistent with TCM, showing apical ballooning and hypokinesia of the mid to apical segments. Interestingly, the base had preserved contractility, and the pattern of hypokinesia extended beyond any single coronary artery territory, leading the diagnosis towards TCM and against myocardial infarction. As the patient was post-thoracotomy, percutaneous coronary intervention was deferred, and anticoagulation/ antiplatelets were held until surgical clearance. The patient continued to be monitored at the Intensive Care Unit (ICU), and dual antiplatelet therapy (DAPT) was planned once the patient was cleared for anticoagulation by the surgery service. The patient eventually developed atrial fibrillation with rapid ventricular response, and amiodarone drip was started. The patient was transferred to a more advanced cardiac center for further monitoring, after which she improved and was discharged stable, with reversion of her atrial fibrillation back to sinus. A follow-up 2D echocardiogram after two months revealed a result similar to pre-operative findings, and the left ventricular dilatation and ballooning were no longer evident (see Figure 1c). The patient’s ejection fraction reverted to normal at 56%. 

## Discussion

TCM is described as a reversible type of heart failure where there are transient regional left ventricular wall motion abnormalities that extend beyond a single epicardial coronary artery territory [7]. It mimics ACS in presentation, with patients usually complaining of chest pain, shortness of breath, or arrhythmias [8]. In severe cases, patients may also present with shock, similar to our case. EKG may show findings such as ST-segment elevations or depressions, T-wave changes, and prolonged QTc intervals [9,10]. Cardiac markers like troponins and pro-BNPs are also commonly elevated, as in our patient. 

2D echocardiography shows a decreased systolic function and ejection fraction, with regional wall motion abnormalities not restricted to one coronary artery territory [7]. In our case, the EF decreased from 60% in the preoperative study to 28% in the postoperative course. The patient was also found to have new-onset mid to apical ballooning, consistent with TCM, as well as hypokinesia in the mid to apical segments. This was further confirmed after doing a cardiac catheterization with ventriculogram, the gold standard in diagnosing TCM. 

The underlying cause and pathophysiology of TCM is not fully understood, but the most widely accepted theory is that it results from sympathetic overstimulation, leading to catecholamine-induced reversible myocardial dysfunction [11]. Vijiiac et al. (2020) list various proposed mechanisms on how the enhanced sympathetic stimulation can induce left ventricular (LV) dysfunction, such as: direct cardiomyocyte toxicity, microvascular dysfunction, multivessel coronary spasm, plaque rupture and thrombosis, and activation of myocardial survival pathways [8]. 

Surgery is one of the triggers of TCM, which occurred in our patient after she underwent esophageal cyst surgery. Immediate post-operative symptoms of heart failure, accompanied by new troponin elevations and echocardiographic findings, strongly allude to the relationship of the stressful event to her cardiomyopathy. 

While TCM is described as a non-occlusive type of cardiomyopathy, co-existent CAD does not exclude the diagnosis [12]. Some studies revealed a high prevalence of vulnerable plaques among patients with TCM [13,14]. A cohort study done by Y-Hassan in 2015 mentioned that ACS can also be a triggering factor in the development of TCM [14], which shows that the coexistence of CAD and TCM is not unlikely. A different study proposes that acute plaque rupture leading to transient ischemia and myocardial stunning was the pathogenic mechanism underlying TCM [13]. Although literature reveals that 15% of patients with TCM have significant atherosclerotic plaques [12], a direct causal association between plaque rupture and TCM requires further investigation. In cardiomyopathies, cardiac manifestations, malignant arrhythmias, and sudden cardiac death dramatically affect prognosis, emphasizing the importance of early diagnosis and a high index of suspicion [15].

Clinical judgment must always be exercised in the approach to TCM, and ACS must always be ruled out, as both cases can present similarly and coexist at the same time. In our patient, cardiac catheterization was done, revealing severe single-vessel CAD with 100% thrombotic occlusion of the large OM3 branch, severe LV dysfunction with LVEF <30%, severely elevated LVEDP of 40 mm Hg, and a left ventriculogram consistent with TCM. Due to the extensive surgery done, cardiothoracic surgery advised against dual antiplatelet therapy temporarily. Losartan was started to prevent cardiac remodeling. Additionally, Atorvastatin was started for CAD. The patient was discharged stable, and follow-up revealed improvement of the ejection fraction, suggesting that the cardiomyopathy was reversible and further confirming TCM as the cause of transient heart failure. Dual antiplatelet therapy was initiated for CAD, and the patient improved to her pre-surgical condition. 

## Conclusions

Diagnosing TCM in patients with coexisting CAD is complex due to the significant overlap with ACS. Both conditions can present with chest pain, ECG changes, elevated troponin, and RWMA, but TCM typically shows RWMA beyond a single coronary artery territory. This clue argues against myocardial infarction, where the wall motion abnormality remains confined to the vessel affected. When CAD is co-existent, it may falsely point clinicians toward a diagnosis of ACS, potentially leading to unnecessary revascularization and missed recognition of TCM. Because TCM can coexist with obstructive CAD, clinical judgment should incorporate the full context, including emotional or physical triggers and imaging findings that do not align with typical infarct patterns, to ensure accurate diagnosis and appropriate treatment.

Similarly, careful consideration must also always be exercised in ruling out ACS in patients presenting with TCM, to prevent missing life-threatening complications requiring emergent intervention. It is important to note that both entities can coexist, and the diagnosis of one does not rule out the other.
